# Gibbs Adsorption and Zener Pinning Enable Mechanically Robust High‐Performance Bi_2_Te_3_‐Based Thermoelectric Devices

**DOI:** 10.1002/advs.202302688

**Published:** 2023-06-29

**Authors:** Chaohua Zhang, Qiangwen Lai, Wu Wang, Xuyang Zhou, Kailiang Lan, Lipeng Hu, Bowen Cai, Matthias Wuttig, Jiaqing He, Fusheng Liu, Yuan Yu

**Affiliations:** ^1^ College of Materials Science and Engineering Shenzhen Key Laboratory of Special Functional Materials Shenzhen Engineering Laboratory for Advanced Technology of Ceramics Guangdong Research Center for Interfacial Engineering of Functional Materials Institute of Deep Underground Sciences and Green Energy Shenzhen University 518060 Shenzhen P. R. China; ^2^ Department of Physics Southern University of Science and Technology Shenzhen 518055 P. R. China; ^3^ Department of Microstructure Physics and Alloy Design Max‐Planck‐Institut für Eisenforschung GmbH 40237 Düsseldorf Germany; ^4^ Shenzhen Jianju Technology Co. Ltd. 518000 Shenzhen P. R. China; ^5^ Institute of Physics (IA) RWTH Aachen University 52056 Aachen Germany; ^6^ PGI 10 (Green IT) Forschungszentrum Jülich GmbH 52428 Jülich Germany

**Keywords:** grain boundary cluster, mechanical property, metavalent bonding, miniature thermoelectric device, Zener pinning

## Abstract

Bi_2_Te_3_‐based alloys have great market demand in miniaturized thermoelectric (TE) devices for solid‐state refrigeration and power generation. However, their poor mechanical properties increase the fabrication cost and decrease the service durability. Here, this work reports on strengthened mechanical robustness in Bi_2_Te_3_‐based alloys due to thermodynamic Gibbs adsorption and kinetic Zener pinning at grain boundaries enabled by MgB_2_ decomposition. These effects result in much‐refined grain size and twofold enhancement of the compressive strength and Vickers hardness in (Bi_0.5_Sb_1.5_Te_3_)_0.97_(MgB_2_)_0.03_ compared with that of traditional powder‐metallurgy‐derived Bi_0.5_Sb_1.5_Te_3_. High mechanical properties enable excellent cutting machinability in the MgB_2_‐added samples, showing no missing corners or cracks. Moreover, adding MgB_2_ facilitates the simultaneous optimization of electron and phonon transport for enhancing the TE figure of merit (ZT). By further optimizing the Bi/Sb ratio, the sample (Bi_0.4_Sb_1.6_Te_3_)_0.97_(MgB_2_)_0.03_ shows a maximum ZT of ≈1.3 at 350 K and an average ZT of 1.1 within 300–473 K. As a consequence, robust TE devices with an energy conversion efficiency of 4.2% at a temperature difference of 215 K are fabricated. This work paves a new way for enhancing the machinability and durability of TE materials, which is especially promising for miniature devices.

## Graphical abstract

Gibbs adsorption and Zener pinning induced by MgB_2_ decomposition are proven to inhibit grain growth for enhancing mechanical properties and cutting processibility of Bi_2_Te_3_‐based alloys with improved thermoelectric performance. An average figure of merit of 1.1 within 300–473 K can be obtained in (Bi_0.4_Sb_1.6_Te_3_)_0.97_(MgB_2_)_0.03_, and an energy conversion efficiency of 4.2% at a temperature difference of 215 K can be achieved in an eight‐pair device.

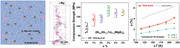

## 1 Introduction

Thermoelectric (TE) materials enable direct transformation between heat and electricity, showing great promise for solid‐state cooling and power generation.^[^ ^1^, ^2^, ^3^, ^4^ ^]^ The performance of a TE material is usually gauged by a dimensionless figure of merit (ZT), defined as $\mathrm{ZT}=S^{2}\sigma T/\kappa$, where *S* is the Seebeck coefficient, *σ* is the electrical conductivity, *T* is the absolute temperature, and *κ* is the total thermal conductivity contributed by the lattice vibration *κ*_lat_ and the carrier transport *κ*_e_.^[^ ^5^ ^]^ Boosting ZT is still the leading goal for TE research, which can be realized by various strategies such as band engineering,^[^ ^6^, ^7^, ^8^ ^]^ microstructural engineering,^[^ ^5^, ^9^, ^10^, ^11^, ^12^, ^13^ ^]^ chemical bonding engineering,^[^ ^14^, ^15^ ^]^ and grain boundary (GB) engineering.^[^ ^16^, ^17^, ^18^ ^]^ Owing to the solid‐state working principle, TE technologies can be utilized for cooling DNA synthesizers, semiconductor lasers, microprocessors, and low‐wattage power generators.^[^ ^1^ ^]^ These applications require miniature TE devices with micron‐scale or even smaller sizes. In this regard, robust mechanical properties are as important as high ZT. Bi_2_Te_3_‐based alloys, the most‐famous TE materials for applications in both solid‐state cooling and power generation near room temperature,^[^ ^10^, ^11^, ^19^, ^20^, ^21^, ^22^, ^23^, ^24^, ^25^, ^26^, ^27^, ^28^, ^29^ ^]^ usually suffer from cracking and failure during device fabrication and service processes. These poor mechanical properties are common features for many chalcogenide thermoelectrics due to the weak and soft metavalent bonds utilized by these compounds.^[^ ^30^, ^31^ ^]^ Metavalent bonding only has a bond order of 0.5 due to the electron deficiency of the bond‐forming p‐states, which can be described as a “two center–one electron” bond.^[^ ^32^ ^]^ Thus, the bond strength is very weak, which is good for low thermal conductivity but not for superior mechanical properties.^[^ ^33^, ^34^ ^]^ The durable application of these compounds requires developing mechanical strengthening strategies to obtain robust TE devices.

The application‐relevant mechanical properties of TE materials mainly consist of hardness, strength and ductility, etc. Generally, introducing different microstructures offers effective routes to enhance mechanical properties. Typical microstructures for strengthening mechanisms include GBs, solid solutions, dislocations, precipitates, and dispersoids.^[^ ^35^, ^36^ ^]^ GB strengthening is the most frequently proposed mechanism, which can be described by the Hall–Petch relationship^[^ ^35^ ^,^ ^37^ ^]^

(1) $$\sigma_{y}=\sigma_{0}\;+k_{y}D^{-1/2}$$

where *σ*_y_ is the yield strength, *σ*_0_ is the friction stress, *k*_y_ is the constant of the Hall–Petch slope, and *D* is the average grain size. Therefore, grain refinement offers a general pathway to enhance mechanical strength.^[^ ^35^, ^37^ ^]^ Yet, the reduction in grain size is often accompanied by an increase in the total Gibbs free energy of the system due to the increased GB fraction and energy.^[^ ^38^ ^]^ Thus, there is a driving force for grain growth to lower the excess interfacial energy. Particularly for TE applications, nanostructured TE materials are often prepared by sintering powder precursors at high temperatures.^[^ ^9^, ^11^, ^39^ ^]^ This will accelerate the coarsening of grains. As a consequence, the high mechanical properties enabled by the small grains can no longer be maintained, which could cause device failure. In addition, grain growth can also enhance the *κ*_lat_ and thus lower ZT.^[^ ^40^, ^41^ ^]^ Therefore, inhibiting grain growth during the sintering process for nanostructured TE materials could provide much‐needed thrust in the wide‐scale application of TE technology.

The propensity of grain growth depends on the GB velocity (*v*), as described by^[^ ^42^ ^]^

(2) $$v\;=M_{\mathrm{GB}}P\;=M_{0}e^{\frac{-Q}{RT}}\frac{2\gamma_{\mathrm{GB}}}{r}$$

where *M*_GB_ is the GB mobility determined by the activation energy for GB migration (*Q*), and *P* is the driving force for grain growth determined by the GB energy (*γ*_GB_) and the principal radius of curvature (*r*). Equation (2) demonstrates that the GB velocity and thus the grain growth can be inhibited by reducing the GB energy thermodynamically and suppressing the GB migration kinetically. For example, stable nanocrystalline W alloys can be designed by alloying with Ti to create heterogeneous solute distributions.^[^ ^43^ ^]^ The segregation of solutes or dopants to interfaces is driven by the decreased Gibbs free energy as described by Gibbs adsorption isotherm.^[^ ^44^ ^]^ The corresponding phenomenon is thus termed Gibbs adsorption, which can lower the GB energy and thus inhibit grain growth. Bulk ultrafine‐grain Fe–22Mn–0.6C steels with high strength and ductility can also be achieved by introducing coherent disordered Cu‐rich precipitates to apply a Zener pinning force to slow down GB migration.^[^ ^45^ ^]^ In thermoelectrics, the grain refinement effect has been frequently reported in several compounds doped with MgB_2_ such as p‐type GeTe^[^ ^46^ ^]^ and n‐type Bi_2_Te_3_‐based alloys,^[^ ^47^ ^]^ as well as p‐type Bi_0.4_Sb_1.6_Te_3_^[^ ^48^ ^]^ and In_0.1_Sb_1.9_Te_3_.^[^ ^49^ ^]^ However, the strengthening mechanisms upon MgB_2_ addition are still elusive and require further investigation.

In this work, as exemplified by MgB_2_‐doped Bi_0.5_Sb_1.5_Te_3_ alloys, we demonstrate a grain refinement effect that stems from both the thermodynamic and kinetic factors due to the decomposition of MgB_2_, leading to strengthened mechanical properties. The segregation of Mg atoms to GBs can lower the GB free energy and thus decrease the driving force for grain growth. On the other hand, the B dispersoids and the Mg‐rich clusters inhibit the GB migration through the Zener pinning effect. As a result, nano‐sized grains can be achieved in the MgB_2_‐added samples even after the high‐temperature sintering process, leading to a twofold improvement in the compressive strength and Vickers hardness in (Bi_0.5_Sb_1.5_Te_3_)_0.97_(MgB_2_)_0.03_. Moreover, the cutting processibility is also greatly enhanced, which is especially promising for miniature TE devices. Those multi‐scale microstructures induced by MgB_2_ decomposition can also reduce the *κ*_lat_ while maintaining the weighted carrier mobility (*µ*_W_), leading to an enhancement of ZT. Further optimizing the Bi/Sb ratio, a higher ZT of 1.3 at 350 K and an average ZT of 1.1 within 300–473 K can be obtained in the sample (Bi_0.4_Sb_1.6_Te_3_)_0.97_(MgB_2_)_0.03_. A robust TE device with an energy conversion efficiency (*η*) of 4.2% at a temperature difference of 215 K using (Bi_0.4_Sb_1.6_Te_3_)_0.97_(MgB_2_)_0.03_ as p‐legs and (Bi_2_Te_2.7_Se_0.3_)_0.995_(MgB_2_)_0.005_ as n‐legs is thus fabricated. Our work unveils the mechanisms underpinning grain refinement by MgB_2_ addition and provides insights into the design of stable nanostructured TE materials and devices by controlling the thermodynamic and kinetic properties of grain boundaries.

## 2 Results and Discussion

### 2.1 Enhancement of Mechanical Properties and Micron‐Scale Cutting Processibility by Adding MgB_2_

Commercial Bi_2_Te_3_‐based alloys are usually prepared by zone‐melting (ZM) methods.^[^ ^22^, ^50^ ^]^ However, owing to the layered structure with weak van der Waals‐like bonding^[^ ^51^, ^52^ ^]^ and the highly preferred orientation, Bi_2_Te_3_‐based ZM ingots tend to cleave along (001) planes, which leads to poor mechanical strength and machinability and, therefore, increasing the cost and failure of TE products.^[^ ^22^, ^24^, ^53^ ^]^ Powder‐metallurgy methods have been widely proven to be effective in enhancing the mechanical performance of Bi_2_Te_3_‐based alloys, mostly owing to the grain refinement‐induced hardening effect.^[^ ^22^, ^54^ ^]^ Typical powder‐metallurgy methods include ball milling (BM),^[^ ^11^ ^]^ solution synthesis^[^ ^55^ ^]^ and melt spinning^[^ ^22^ ^]^ combined with hot pressing or spark plasma sintering (SPS). Our (Bi_0.5_Sb_1.5_Te_3_)_1−_ *_x_* (MgB_2_)*_x_* samples were prepared using the traditional powder‐metallurgy method that includes melting, ball‐milling, and SPS processes.

**Figure 1:**
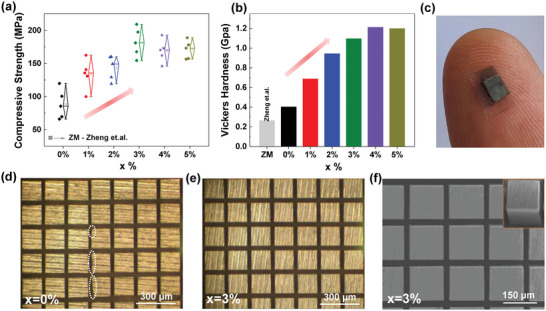
Enhanced mechanical properties and cutting processibility by adding MgB_2_. a) Compressive strength and b) Vickers hardness of (Bi_0.5_Sb_1.5_Te_3_)_1−_ *_x_* (MgB_2_)*_x_* with different addition of MgB_2_. The literature data of zone‐melting (ZM) ingots are also listed for comparison.^[^ ^22^ ^]^ c) Photograph of the *x* = 3% sample supported on an adhesive tape after micron‐scale cutting, laying on a finger. Optical microscopy images of the d) *x* = 0% and e) *x* = 3% samples after cutting. f) Scanning electron microscopy (SEM) image of the *x* = 3% samples after cutting, and the section view of a micronized bar is also displayed in the inset.

As shown in **Figure** **1**a,b, compared with traditional ZM ingots,^[^ ^22^ ^]^ the compressive strength and Vickers hardness of our MgB_2_‐free powder‐metallurgy‐processed Bi_0.5_Sb_1.5_Te_3_ can be enhanced from ≈37 to ≈88 MPa, and from ≈0.26 GPa to ≈0.40 GPa, respectively. The mechanical properties of our MgB_2_‐free sample (*x* = 0%) are at the same level as the melt‐spun‐sintering processed Bi_0.5_Sb_1.5_Te_3_.^[^ ^22^ ^]^ By introducing MgB_2_, the compressive strength and Vickers hardness of our (Bi_0.5_Sb_1.5_Te_3_)_1−_ *_x_* (MgB_2_)*_x_* samples can be further enhanced. The highest average compressive strength can reach 182 MPa for the *x* = 3% sample, which is more than twice that of the *x* = 0% sample. The highest hardness can reach ≈1.2 GPa for the *x* = 4% sample, which is around three times that of the *x* = 0% sample.

From the view of commercial applications, machinability testing provides a direct way to check the mechanical strength of Bi_2_Te_3_‐based alloys. Our bulk samples were firstly cut into thin chips with a thickness of ≈200 µm, and then these thin chips were attached on an adhesive tape for further cutting into small bars with a cross‐section of ≈300 × 300 µm or ≈150 × 150 µm, as illustrated in Figure 1c. We found that the mechanical strengthening by powder‐metallurgy treatment is good enough to realize perfect small bars with the size of ≈300 × 300 × 200 µm for the MgB_2_‐free sample (*x* = 0%), showing no cracks or missing corners with a yield of ≈100% (Figure S1, Supporting Information). However, when the *x* = 0% sample was cut into smaller bars with the size of ≈150 × 150 × 200 µm, many obvious missing corners and cracks (Figure 1d; Figure S2, Supporting Information) can be observed, and the yield can only reach ≈92% (Figure S2, Supporting Information). Therefore, the mechanical strengthening by the traditional powder‐metallurgy method is not good enough for fabricating miniature devices with much smaller TE legs. Note that the zone‐melting prepared ingots cannot be cut into such a small shape. In stark contrast, after cutting into smaller bars with the size of ≈150 × 150 × 200 µm, the MgB_2_‐added sample with *x* = 3% can still maintain the perfect bar shape without missing corners or cracks with a yield of ≈100% (Figure 1e,f; Figure S3, Supporting Information). Such excellent mechanical properties and micron‐scale cutting processibility are especially attractive for developing miniature TE devices at low cost.

### 2.2 Grain Refinement by Adding MgB_2_

**Figure 2:**
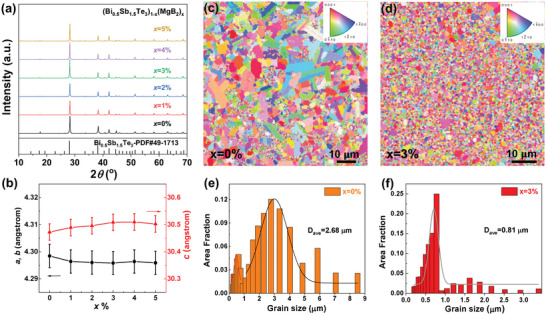
Effects of MgB_2_ on lattice parameters and grain boundaries. a) X‐ray diffraction (XRD) patterns and b) corresponding lattice parameters of the (Bi_0.5_Sb_1.5_Te_3_)_1−_ *_x_* (MgB_2_)*_x_*. Electron backscatter diffraction (EBSD) images of the (Bi_0.5_Sb_1.5_Te_3_)_1−_ *_x_* (MgB_2_)*_x_* samples with c) *x* = 0% and d) *x* = 3%, and the corresponding grain‐size distributions for the samples with e) *x* = 0% and f) *x* = 3%. Error bars of 0.1% in b) are set.

To reveal the mechanisms of the great enhancement of mechanical properties and cutting processibility by adding MgB_2_ in Bi_2_Te_3_, we carried out multi‐scale microstructure characterizations. **Figure** **2**a shows the X‐ray diffraction (XRD) patterns of (Bi_0.5_Sb_1.5_Te_3_)_1−_ *_x_* (MgB_2_)*_x_* samples after SPS, which can be indexed to the standard PDF card of Bi_0.5_Sb_1.5_Te_3_ (PDF#49‐1713, *R‐3m* space group). As shown in Figure 2b, the lattice parameters *a, b* and *c* are calculated by the Rietveld refinement of the XRD patterns in Figure 2a. The lattice parameter *a* (*b = a*) and *c* are nearly independent of MgB_2_ content in the error range of 0.1%, though a slight decrease tendency in *a* and increase in *c* can be identified when increasing MgB_2_ content from *x* = 0% to 2%. The nearly unchanged *a* (*b = a*) and *c* by adding MgB_2_ indicate that Mg and B elements may have quite limited solid solubility in Bi_0.5_Sb_1.5_Te_3_, suggesting that the addition of MgB_2_ mainly impacts the GB environment.

As shown in Figure 1c–f, adding MgB_2_ can result in an obvious grain refinement effect in (Bi_0.5_Sb_1.5_Te_3_)_1−_ *_x_* (MgB_2_)*_x_*, as demonstrated by the electron backscatter diffraction (EBSD) characterizations. The MgB_2_‐free sample with *x* = 0% shows a broad grain‐size distribution up to 8.5 µm, having an average grain size of ≈2.7 µm. In stark contrast, the MgB_2_‐added sample with *x* = 3% displays grain sizes mostly below 1 µm, though sporadic grain size distribution from 1 to 3.4 µm can also be displayed. Corresponding average grain size can be as low as ≈0.81 µm for (Bi_0.5_Sb_1.5_Te_3_)_0.97_(MgB_2_)_0.03_, more than three times smaller than the MgB_2_‐free sample. The grain refinement can also be demonstrated by observing the fracture morphology of the (Bi_0.5_Sb_1.5_Te_3_)_1−_ *_x_* (MgB_2_)*_x_* alloys (Figure S4, Supporting Information). According to the Hall–Petch relationship shown in Equation (1), grain refinement should be an important factor for the enhanced mechanical properties in MgB_2_‐added samples. The reasons for the strikingly refined grains in the MgB_2_‐added samples are crucial for the materials design and will be studied by comprehensive structural characterizations below.

### 2.3 Microstructure Characterizations by SEM, AES, STEM, and APT

**Figure 3:**
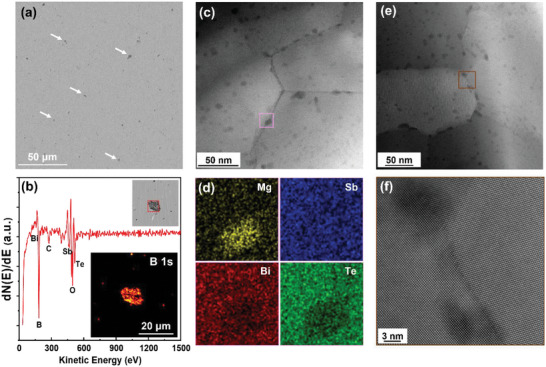
Microstructure characterizations of the sample (Bi_0.5_Sb_1.5_Te_3_)_1−_ *_x_* (MgB_2_)*_x_* with *x* = 3% by SEM, AES and STEM. a) Low‐magnification SEM image showing various dark particles, as indicated by arrows. b) Auger electron spectroscopy (AES) collected in a red‐dot‐square region shown in the inset. The corresponding AES elemental mapping image of B1s is also displayed in the inset. c) Overview of HAADF‐STEM image, showing abundant nanoclusters both within grains and along grain boundaries. d) The EDS‐STEM elemental mappings of Mg, Sb, Bi, and Te for the marked region in (c) that includes a nanocluster along the grain boundary. e) Typical HAADF‐STEM image in a region with different grain orientations. f) High‐resolution STEM image of nanoclusters near a grain boundary marked in (e).

**Figure** **3**a shows many sporadically dispersed micro‐level particles with dark contrast as indicated by arrows. We performed the Auger electron spectroscopy (AES) characterizations on some regions with obviously bigger particles, as shown in the inset of Figure 3b. As the AES is much more sensitive to light elements, the B element shows much stronger signals than the heavy elements such as Bi, Sb and Te. However, the Mg signal is not detected by AES owing to its very low concentration. The detected C and O signals in Figure 3b should be ascribed to the frequently observed organic contaminant on the surface. As shown in the inset of Figure 3b, the AES elemental mapping of B1s demonstrates that those micro‐level particles observed in SEM (Figure 3a) are segregated B dispersoids.

Scanning transmission electron microscopy (STEM) with high‐angle annular dark‐field (HAADF) imaging was applied to analyze the nano and microstructures of Bi_2_Te_3_‐based alloys induced by adding MgB_2_, where the contrast of HAADF‐STEM image is roughly proportional to the square of atomic number in the specimen. As displayed in the overview HAADF‐STEM images, abundant nanocluster‐like features with dark contrasts to the matrix can be observed in the (Bi_0.5_Sb_1.5_Te_3_)_1−_ *_x_* (MgB_2_)*_x_* samples with *x* = 3% (Figure 3c,e) and *x* = 5% (Figures S5 and S6, Supporting Information). These nanoclusters with a size of around 5–20 nm distribute at both GBs and within grains, where the dark contrast of nanoclusters in the HAADF image could indicate the existence of light‐element compared to the matrix. Energy‐dispersive X‐ray spectroscopy (EDS) in STEM was further applied to investigate the composition of these nanoclusters (Figure 3d; Figure S6, Supporting Information), showing Mg‐rich characteristics. Therefore, the observation of separated Mg by STEM and B by AES indicates the decomposition of MgB_2_ during the preparation process. We also take a high‐resolution STEM image of the nanoclusters near a grain boundary (Figure 3f), showing nearly the same lattice patterns as other regions without nanoclusters. This indicates that the lattice of nanoclusters is coherent with the matrix. It is also energetically favorable to form coherent structures with the matrix for an extremely small second phase to minimize interfacial elastic energy.

**Figure 4:**
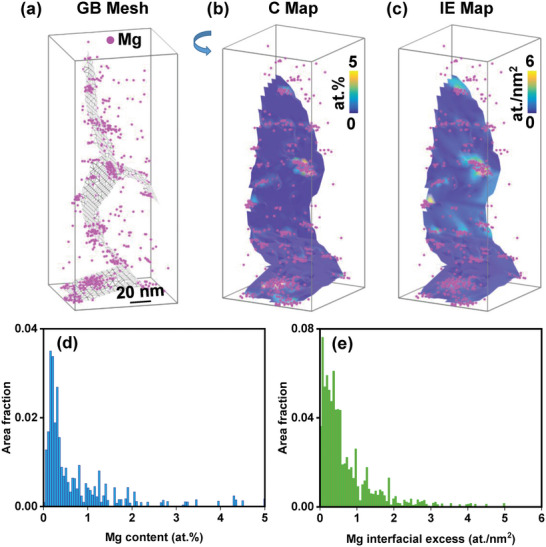
Microstructure characterizations of (Bi_0.5_Sb_1.5_Te_3_)_0.97_(MgB_2_)_0.03_ by atom probe tomography (APT). a) The atom map shows Mg embedded with the meshed GB plane. The mesh of the GB plane was created by recognizing GB traces using a convolutional neural network.^[^ ^57^ ^]^ b) The composition map and c) the interfacial excess map illustrate the distribution of solute Mg along the GB plane shown in (a). The solute Mg atoms are distributed along the GB planes of the (Bi_0.5_Sb_1.5_Te_3_)_0.97_(MgB_2_)_0.03_ alloy, represented by the area fraction of a given Mg content as a function of d) Mg composition and e) Mg interfacial excess.

To better resolve the spatial distribution of Mg and possibly B, atom probe tomography (APT) characterizations were carried out.^[^ ^56^ ^]^ We analyzed the APT data by the method developed by Zhou et al.,^[^ ^57^ ^]^ which can reveal the in‐plane chemical features and the Gibbsian interfacial excess that could not be identified by standard compositional analyses. **Figure** **4**a shows the GB mesh created by recognizing GB traces using a convolutional neural network. This meshing enables a more accurate calculation of the GB compositions independent of the local curvature of GBs. Figure 4b displays the local composition of Mg within the GB planes. Dispersed Mg‐rich clusters can be discerned with a maximum concentration of about 5 at. % Mg located on the GB, which is consistent with the nanoclusters observed in STEM. We also calculated the Gibbsian interfacial excess value of Mg within the GBs, as demonstrated in Figure 4c. This value is independent of the GB thickness and can circumvent the artifacts induced by the local magnification effect at GBs in APT measurements.^[^ ^58^ ^]^ The positive or negative interfacial excess is also indicative of the Gibbs adsorption or desorption of impurities, which impacts the GB free energy and thus the driving force for grain growth. This will be further discussed in the next section. Figure 4d,e illustrates the area fraction of Mg composition and interfacial excess within the GB planes, respectively. The majority of the GBs are covered by Mg atoms with a very low Mg content and a small excess. These Mg atoms cover the GB plane by Gibbs adsorption impacting the interfacial free energy thermodynamically. Yet, we also observed some dispersed areas with a high content of Mg and a large interfacial excess. These parts should be assigned to Mg‐rich clusters, which will slow the GB mobility kinetically. APT also confirms the negligible solubility of Mg and B in the matrix, which is consistent with the XRD results. This is critical for the segregation of Mg and B to GBs and the formation of Mg‐rich clusters and B dispersoids. The limited solubility of dopants is in line with the design rules for forming GB complexions and clusters as discussed by An et al.^[^ ^59^ ^]^ Besides the factors of atomic size and electronegativity differences, the chemical bonding mechanisms also influence the solubility of dopants.^[^ ^60^ ^]^ For metavalently bonded compounds such as bismuth telluride, the dopants should utilize other bonding mechanisms such as found for MgB_2_ and SiC to promote segregation and clustering. More suitable combinations for matrix and dopants can be found in a treasure map provided in our previous work.^[^ ^15^, ^60^ ^]^

### 2.4 Mechanisms of Gibbs Adsorption and Zener Pinning on Reducing the Grain Size

Based on the above microstructural characterization, the formation of Mg‐rich GB complexions from Gibbs adsorption and nanoclusters as well as B dispersoids in Bi–Sb–Te alloys can be derived from the decomposition of MgB_2_ during the sample preparation process (i.e., melting, ball milling and SPS processes), as described in the following reaction equation

(3) $$\left(\mathrm{Sb},\mathrm{Bi},\mathrm{Te}\right)+\mathrm{xMg}B_{2}\to {\left(\mathrm{xMg},\mathrm{Sb},\mathrm{Bi},\mathrm{Te}\right)}_{\mathrm{cluster}/\mathrm{complexion}}+2xB_{\mathrm{dispersoid}}$$

**Figure 5:**
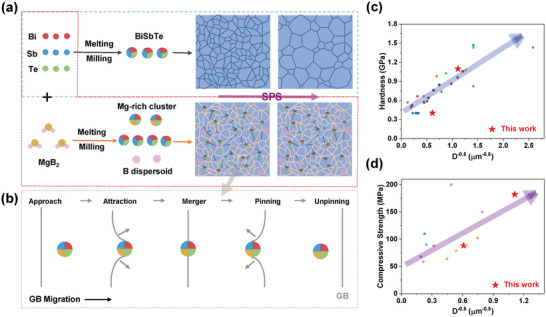
Mechanisms analysis. a) Grain‐size evolutions with and without adding MgB_2_ during the sample preparation processes. Besides inducing Mg‐rich cluster and B dispersoid when adding MgB_2_, Mg‐rich GB complexions (colored GBs) can also be formed due to Gibbs adsorption. b) Typical states for GB–particle interactions during the GB migration process: approach, attraction, merger, pinning, and unpinning. Unpinning the particles from GBs then requires a stronger driving force to overcome the Zener pinning force, which impedes grain growth. Grain‐size dependent c) hardness and d) compressive strength based on this work and other reported data of hardness^[^ ^22^, ^54^, ^64^, ^65^, ^66^, ^67^, ^68^, ^69^ ^]^ and compressive strength,^[^ ^22^, ^54^, ^69^, ^70^, ^71^ ^]^ mostly follow the Hall–Petch rule.

As shown in **Figure** **5**a, for the MgB_2_‐free Bi–Sb–Te alloys, grain growth can be initiated easily driven by the high temperature and high pressure during the SPS process. However, for the MgB_2_‐added samples, the decomposition‐derived Mg‐rich GB complexions and nanoclusters as well as B dispersoids in Bi–Sb–Te alloys can thermodynamically and kinetically impede the grain growth during the SPS process, leading to the grain refinement phenomenon (Figure 2).

Usually, the hindrance of grain growth by second‐phase particles or dispersoids is understood by the Zener pinning effect,^[^ ^61^, ^62^, ^63^ ^]^ as illustrated in Figure 5b being exampled by our Mg‐rich clusters. Besides the Mg‐rich clusters, the B dispersoids observed in this work can also serve as “particles” for Zener pinning. The GB–particle interaction has two main states: attraction and pinning (Figure 5b). As soon as the migrating GB approaches particles and gets touched, the surface tension begins to pull the GB and particle toward each other to reduce the Gibbs energy. This explains why most of these Mg‐rich clusters are attracted to GBs. However, when the GB–particle system reaches the minimum energy state, extra energy is needed to restore the GB by further moving off these clusters from GB. That is to say, unpinning the particles from GBs requires a stronger driving force to overcome the pinning force. Generally, the Zener pinning pressure *P_Z_* (pinning force exerted on a unit area of GB) for impeding grain growth can be approximated as^[^ ^61^, ^62^ ^]^

(4) $$P_{Z}\propto \frac{f_{V}\gamma_{\mathrm{GB}}}{r}$$

where *r* and *f*_V_ are the radius and volume fraction of the dispersed particles, respectively. Therefore, smaller particles in high fractions are more favorable for grain refinement. Moreover, based on our STEM and APT observations, we can infer that those Mg‐rich nanoclusters should be the main contributor to the pinning force, though larger‐size B dispersoids can also contribute to part of the pinning effects.

Besides the Zener pinning from GB clusters, APT results also show Gibbs adsorption of Mg to the GBs, forming Mg‐rich GB complexions, which leads to a positive interfacial excess as demonstrated in Figure 4e. According to the Gibbs adsorption isotherm and McLean's GB segregation model, the GB energy *γ*_GB_ can be expressed as^[^ ^59^ ^]^

(5) $$\gamma_{\mathrm{GB}}=\gamma_{0}\;-\Gamma \left(RT\ln X+\Delta H^{\mathrm{seg}}\right)$$

where *γ*_0_ is the pure GB energy, *R* is the gas constant, *T* is the annealing temperature, *X* is the dopant content in the matrix, and Δ*H* ^seg^ is the segregation enthalpy. *Γ* is the Gibbssian interfacial excess, which can be obtained in Figure 4e. The positive *Γ* value indicates that the GB energy can be lowered due to the Mg‐rich GB complexions. This will reduce the driving force for grain growth as described in Equation (2). A similar effect has also been observed to retard the Ostwald ripening process of nanoprecipitates.^[^ ^59^ ^]^ Therefore, the much‐refined grains in our work upon adding MgB_2_ should be attributed to the synergetic effects of thermodynamic Gibbs adsorption and kinetic Zener pinning.

According to the Hall–Petch relationship (Equation (1)), we plot the *D* ^−1/2^ dependent hardness (Figure 5c) and compressive strength (Figure 5d) of Bi_2_Te_3_‐based alloys based on this work and other reported hardness^[^ ^22^, ^54^, ^64^, ^65^, ^66^, ^67^, ^68^, ^69^ ^]^ and compressive strength.^[^ ^22^, ^54^, ^69^, ^70^, ^71^ ^]^ Although the compositions and preparation methods of these Bi_2_Te_3_‐based samples are quite different, their mechanical strength mostly follows the Hall–Petch rule when ignoring some measurement errors. Therefore, GB strengthening by grain refinement is the leading mechanism for enhancing the mechanical performance of Bi_2_Te_3_‐based alloys. Besides, Mg‐rich nanoclusters and B dispersoids may also impede the motion of dislocations, therefore bringing in extra strengthening mechanisms, i.e., precipitate and dispersoid strengthening. Our work suggests that introducing Zener pinning effects for grain refinement can be a general method to enhance mechanical properties, which should also be applied in other B or MgB_2_‐doped GeTe^[^ ^46^, ^72^ ^]^ and Bi_2_Te_3_.^[^ ^47^, ^48^, ^49^, ^54^ ^]^ Moreover, the enhanced mechanical strength of Bi_2_Te_3_‐based alloys by introducing SiC,^[^ ^23^, ^73^ ^]^ MoS_2_,^[^ ^65^ ^]^ and TiC^[^ ^68^ ^]^ nanodispersions should also be ascribed to the Zener pinning effects.

### 2.5 Enhancement of Thermoelectric Properties by Adding MgB_2_ and Tuning the Bi/Sb Ratio

**Figure 6:**
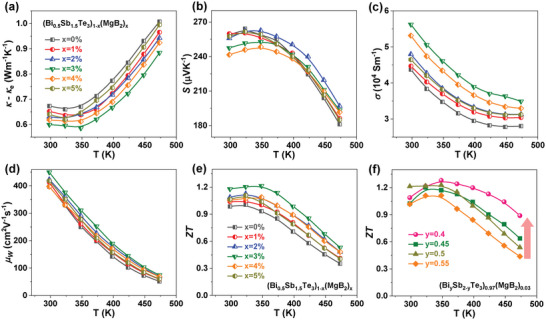
Thermoelectric properties. Temperature‐dependent a) lattice thermal conductivity *κ*–*κ*_e_, b) Seebeck coefficient *S* c) electrical conductivity *σ*, d) weighted mobility *µ*_w_ and e) ZT of (Bi_0.5_Sb_1.5_Te_3_)_1−_ *_x_* (MgB_2_)*_x_* with different addition of MgB_2_. f) Temperature‐dependent ZT of (Bi_y_Sb_2−y_Te_3_)_0.97_(MgB_2_)_0.03_ with different Bi/Sb ratio. Panels (a)–(e) share the same notation.

Besides the mechanical properties, TE properties were also evaluated to reveal the effects of adding MgB_2_, as shown in **Figure** **6**. The refined grain sizes and the GB clusters and dispersoids enhance the phonon scattering, leading to a reduced *κ*_lat_, as illustrated in Figure 6a. The increase of *κ*_lat_ at higher MgB_2_ content (*x* > 3%) should be ascribed to the intrinsic high *κ* from segregated B dispersoids, which have also been observed in B‐added GeTe and Bi_2_Te_3_.^[^ ^54^, ^72^ ^]^ By increasing the MgB_2_ content, the *S* displays a minor irregular variation in the error range of ≈5% (Figure 6b). This phenomenon agrees well with the nearly ignorable solid solubility of Mg and B in the Bi_2_Te_3_‐based matrix (Figure 2b), which leads to a quite limited influence on carrier density and effective mass (Figure S7, Supporting Information). In stark contrast, the *σ* shows much obvious dependence on MgB_2_, which increases firstly and then decreases with increasing MgB_2_ content (Figure 6c), reaching the highest values for the *x* = 3% sample. The simultaneous decrease in *κ*_lat_ and the increase in *σ* is striking. This should be attributed to the coherent nature of GB clusters with the matrix and the formation of Mg‐rich GB complexions. Further increasing the content of MgB_2_ (*x* > 3%) could induce the formation of GB precipitates, which will lower the total *σ*, as observed in Ga‐doped GeTe.^[^ ^17^ ^]^ The increased *σ* and maintained *S* lead to enhanced weighted mobility (*µ*_W_),^[^ ^74^ ^]^ demonstrating the improved electronic transport properties of Bi_2_Te_3_ by adding MgB_2_ (Figure 6d). Therefore, enhanced ZT values can be obtained in the MgB_2_‐added samples, where the maximum reaches in sample *x* = 3% (Figure 6e).

Based on the beneficial effect of MgB_2_ on decoupling the electron and phonon transport, we further tune the Bi/Sb ratio of p‐type (Bi,Sb)_2_Te_3_‐based alloys (Figure S8, Supporting Information) for optimizing their carrier density, band structures, and bipolar transport, which have been widely used in previous reports.^[^ ^24^, ^26^ ^]^ Usually, the strong bipolar transport in Bi_2_Te_3_ can lead to a significant increase of *κ*_lat_ at higher temperatures (Figure 6a), which can be suppressed by optimizing the Bi/Sb ratio (Figure S8, Supporting Information). Therefore, owing to the optimization of carrier density and suppressed bipolar transport, the optimal (Bi_0.4_Sb_1.6_Te_3_)_0.97_(MgB_2_)_0.03_ sample exhibits the highest ZT of ≈1.3 at 350 K and an average ZT of 1.1 within 300–473 K (Figure 6f), which are higher than the corresponding commercial ZM ingots and comparable to other reports.^[^ ^22^, ^73^ ^]^ The enhancement of ZT at higher temperatures (>350 K) for the (Bi_0.4_Sb_1.6_Te_3_)_0.97_(MgB_2_)_0.03_ sample makes it attractive for power generation. We also measured the Vickers hardness of the sintered (Bi_0.4_Sb_1.6_Te_3_)_0.97_(MgB_2_)_0.03_ pellet taken from different locations, demonstrating robust mechanical properties with good uniformity (Figure S9, Supporting Information).

### 2.6 Robust Thermoelectric Devices Based on MgB_2_‐Modified Bi_2_Te_3_‐Based Alloys

**Figure 7:**
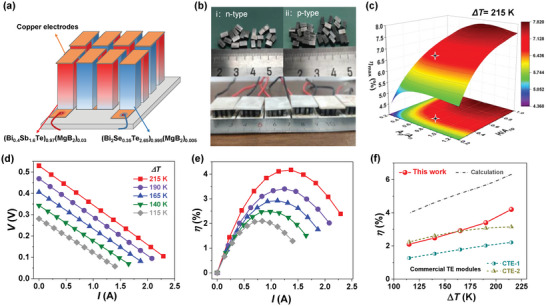
Thermoelectric devices based on MgB_2_‐modified Bi_2_Te_3_‐based alloys. a) Schematic structure of an eight‐pair (Bi_0.4_Sb_1.6_Te_3_)_0.97_(MgB_2_)_0.03_/(Bi_2_Te_2.65_Se_0.35_)_0.995_(MgB_2_)_0.005_ TE device. b) The n/p‐type TE legs after cutting and the optical image of our assembled TE devices. c) Theoretical modeling of the maximum energy conversion efficiency (*η*) at temperature differences (Δ*T*) of 215 K (cold‐site temperature fixed at 283 K), where the section area ratio of p‐type versus n‐type leg (*A*_p_/*A*_n_) and the ratio of leg height versus total section area (*H*/*A*_np_) are varied. Current‐dependent d) output voltage and e) *η* of the TE device under different Δ*T*. f) The *η* of our TE device under different Δ*T*, which is compared with two commercial TE modules and calculated values in (c).

As demonstrated in our previous report, MgB_2_ can also simultaneously enhance the mechanical and TE performance of n‐type Bi_2_Te_3_‐based alloys.^[^ ^47^ ^]^ As shown in **Figure** **7**a, to further demonstrate the MgB_2_‐doped Bi_2_Te_3_ samples for practical applications, the MgB_2_‐modified n‐type (Bi_2_Te_2.65_Se_0.35_)_0.995_(MgB_2_)_0.005_ (TE properties listed in Figure S10 in the Supporting Information) and p‐type (Bi_0.4_Sb_1.6_Te_3_)_0.97_(MgB_2_)_0.03_ are used to fabricate the eight‐pair TE devices. The device performance is measured by a commercial testing system (PEM‐2), wherein the cold‐site temperature is fixed at 283 K and the hot‐side temperature is varied to obtain various temperature differences (Δ*T*). As references, two commercial TE modules were bought from different companies and were also tested by our equipment. As shown in Figure 7b, the strong mechanical strength of our MgB_2_‐modified Bi_2_Te_3_‐based alloys can enable good cutting processibility without the annoying cracking problem, which then enables easy assembling of TE devices.

Besides the thermoelectric properties of TE legs, the size of TE legs can also influence the conversion efficiency (*η*) (Figure 7c) and the output power *P* (Figure S11 Supporting Information). Theoretically, at the fixed section area ratio of p‐type versus n‐type leg (*A*_p_/*A*_n_), increasing the ratio of leg height versus total section area (*H*/*A*_np_) can increase the *η* (Figure 7c) but decrease the *P* (Figure S11, Supporting Information). Therefore, as highlighted in Figure 7c, compromised *H*/*A*_np_ (≈0.34 mm^−1^) and optimized *A*_p_/*A*_n_ (≈0.82) are adopted in our actual TE devices to simultaneously obtain good *η* and *P*. As shown in Figure 7d, the open‐circuit voltage (*V*_OC_) increases from 0.28 to 0.53 V with the Δ*T* increasing from 90 to 215 K, and the output voltage *V* declines linearly with the increase of current *I*. Therefore, *η* (Figure 7e) at specific Δ*T* both gradually increase to peak values and then decline with the increase of *I*. The optimal current for peak *η* at specific Δ*T* is obtained when the external electrical load is identical to internal resistance *R*_in_. As a result, a maximum *η* of 4.2% at Δ*T* = 215 K and *I* = 1.37 A can be achieved. Considering the radiant heat loss during the measurement,^[^ ^72^ ^]^ the *η* of our TE devices is somehow underestimated (Figure S12, Supporting Information). This problem has also been discussed in other literature^[^ ^75^, ^76^ ^]^ and our previous report.^[^ ^72^ ^]^ Moreover, determining the heat flow by different homemade setups can also raise the uncertainty of *η*. In the absence of a heat loss problem, our theoretical modeling predicts a *η* of 6.2% at Δ*T* = 215 K (Figure 7f; Figure S12, Supporting Information), which is in line with many advanced Bi_2_Te_3_‐based TE devices.^[^ ^29^, ^77^ ^]^ To enable a direct comparison with commercial products, we purchased two commercial TE devices from different companies and measured their *η* using our PEM‐2 instrument (Figure S13, Supporting Information). As shown in Figure 7f, the *η* of our TE devices is higher (>175 K) and comparable with commercial TE devices, showing a great advantage of our MgB_2_‐modified Bi_2_Te_3_‐based alloys with much‐improved stability for commercial use.

## 3 Conclusion

In summary, we reported on a mechanical strengthening strategy enabled by MgB_2_ decomposition in Bi_2_Te_3_‐based alloys. The decomposed Mg and B elements show negligible solubility in the lattice of Bi_2_Te_3_. They segregate to grain boundaries to partly decorate the GB‐forming complexions and partly aggregate as Mg‐rich GB clusters and B dispersoids. These microstructures inhibit grain growth due to the reduced GB energy thermodynamically and the increased Zener pinning dragging forces kinetically. Therefore, the compressive strength and Vickers hardness of (Bi_0.5_Sb_1.5_Te_3_)_1−_ *_x_* (MgB_2_)*_x_* with *x* = 3% can be enhanced by more than two times, compared with the MgB_2_‐free Bi_0.5_Sb_1.5_Te_3_ prepared by traditional BM‐SPS method. The MgB_2_‐added sample also shows excellent micron‐scale cutting processibility, which can be cut into smaller bars with the size of ≈150 × 150 × 200 µm without any missing corners or cracks. Moreover, the introduced microstructures by adding MgB_2_ can also decouple the electron and phonon transport, simultaneously realizing increased *µ*_W_ and decreased *κ*_lat_ for enhancing the ZT. The ZT can be further enhanced by optimizing the Bi/Sb ratio. The optimal (Bi_0.4_Sb_1.6_Te_3_)_0.97_(MgB_2_)_0.03_ sample shows the highest ZT of ≈1.3 at 350 K and an average ZT of 1.1 within 300–473 K. A robust TE device using (Bi_0.4_Sb_1.6_Te_3_)_0.97_(MgB_2_)_0.03_ as p‐leg and (Bi_2_Te_2.7_Se_0.3_)_0.995_(MgB_2_)_0.005_ as n‐leg is fabricated, obtaining an energy conversion efficiency of 4.2% at a temperature difference of 215 K. This strategy of adding MgB_2_ paves a new way for enhancing the mechanical properties and cutting processibility of TE materials, which is especially attractive for miniature devices in practical applications.

## 4 Experimental Section

### Materials Synthesis

To prepare (Bi_0.5_Sb_1.5_Te_3_)_1−_ *_x_* (MgB_2_)*_x_* and (Bi*_x_* Sb_2−_ *_x_* Te_3_)_0.97_(MgB_2_)_0.03_ samples, the precursors of Bi (99.99%), Te (99.99%), Sb (99.99%), and MgB_2_ (99.999%) were weighed and sealed in the evacuated quartz tube (3×10^−3^ Pa). The raw materials were heated at 950 °C for 16 h and then slowly cooled down to 600 °C in 10 h. After holding at 600 °C for 10 h, the obtained ingots were ground into powders by ball milling, and then the powders were sintered into bulk pellets with a diameter of ≈20 mm and thickness of ≈4 mm using spark plasma sintering (SPS) process (500 °C, 5 min; 60 MPa).

### Materials Characterizations

XRD characterizations were performed using a Bruker D8 advance SS/18 kW diffractometer with Cu *Kα* radiation (*λ* = 0.15 404 nm; 40 kV, 200 mA). SEM (Hitachi SU‐70) equipped with EDS and AES (PHI 710) were used to analyze the morphology and elemental distribution. For the EBSD analysis (EDAX‐TSL, USA), the samples were further polished by a vibration polisher (Vibromet2, Buehler), and then were characterized at a given tilt angle of 70° with an accelerating voltage of 20 kV and a step size 0.5 µm. Scanning transmission electron microscopy (STEM) measurements of the samples were performed on a double Cs‐corrected TEM (FEI Titan Themis G2) with a Super‐X EDS detector, operated at 300 kV. Needle‐shaped APT specimens were prepared by a dual‐beam scanning electron microscopy/focused ion beam (SEM/FIB) (Helios NanoLab 650, FEI, USA) following the standard “lift‐out” method. APT measurements were conducted on a local electrode atom probe (LEAP 4000X Si, Cameca, USA). The laser pulses were adapted with a wavelength of 355 nm, a pulse duration of 10 ps, and pulse energy of 20 pJ. A pulse repetition rate of 200 kHz with a detection rate of 1% on average, an ion flight path of 160 mm, and a specimen base temperature of 40 K were utilized. The APT data were processed using the commercial software package IVAS 3.8.0 from Cameca Instruments.

### Thermoelectric Properties

The sintered samples were cut into 4.0 mm × 4.0 mm × 13.0 mm for the measurement of electrical properties (*S*, *σ*), using a ZEM‐3 apparatus (Ulvac‐Riko) under a helium atmosphere. The sintered samples were cut into 10 mm × 10 mm × 2.1 mm for measuring the thermal diffusivity (*λ*) parallel to the press direction, using the laser flash equipment (LFA‐467, NETZSCH). To measure the *λ* along the same direction with *S* and *σ*, we adopted a “cutting–rotating–pasting” method, which was proven to be reliable with a negligible influence of the glue on the measured *λ*.^[^ ^39^ ^]^ Besides *λ*, the density *d* and specific heat capacity *C*_p_ were also needed to obtain the final thermal conductivity *κ* (=*λdC*_p_), where *d* was measured by the Archimedes method and the *C*_p_ was calculated by the Dulong–Petit law. The *κ*_lat_ is calculated by subtracting *κ*_e_ from *κ*, *κ*_lat_ =  *κ* −  *κ*_e_ =  *κ* − *LσT*, where *L* is the Lorenz number. The *L* is estimated by an empirical formula,^[^ ^78^ ^]^ *L*  =  1.5 + exp( − |*S*|/116), where *S* is in the unit of µV K^−1^ and *L* in 10^−8^ W Ω K^−2^. The Vickers hardness measurement was conducted by applying a force of 0.49 N and maintained for 5 s on a hardness tester (HX‐1000TMC, Shanghai Taiming, China). The compressive strength was measured based on a universal testing machine (CMT5105).

### Thermoelectric Device Fabrication and Characterization

Using the traditional electroplating method, Ni as an interfacial barrier layer was coated on the SPS‐derived samples for fabricating (Bi_0.4_Sb_1.6_Te_3_)_0.97_(MgB_2_)_0.03_/Ni and (Bi_2_Te_2.65_Se_0.35_)_0.995_(MgB_2_)_0.005_/Ni TE legs. The p‐type and n‐type TE legs were cut into the geometric dimensions of ≈2.7 mm × 2.3 mm × 4.6 mm and ≈2.7 mm × 2.8 mm × 4.6 mm, respectively. The Sn_64_Bi_35_Ag_1_ solder paste was used to connect the TE legs with the copper plates. The purchased commercial devices of CET‐1 and CET‐2 were 127‐pair thermoelectric modules with dimensions of 40 mm × 40 mm × 3.8 mm. The output performance of TE devices was measured by a commercial TE‐module testing system (Advance Riko, PEM‐2) in a helium atmosphere. The cold site temperature *T_c_* was maintained at 283 K by a recycled cooling setup, and then the hot site temperature *T*_h_ was increased to corresponding temperatures for the device test. As the platform size of the commercial PEM‐2 (40 mm × 40 mm) was much larger than the size of the eight‐couple TE module, the energy conversion efficiency of the eight‐couple TE module was greatly underestimated due to the existence of radiant heat. The use of a silica‐aerogel blanker as a heat insulator can substantially reduce the radiant heat, which alleviates the measurement errors of conversion efficiency.

## Conflict of Interest

The authors declare no conflict of interest.

## Supporting information

Supporting Information

## Data Availability

The data that support the findings of this study are available from the corresponding author upon reasonable request.
