# Supplementary material for: Gibbs Adsorption and Zener Pinning Enable Mechanically Robust High‐Performance Bi2Te3‐Based Thermoelectric Devices
Source: Adv Sci (Weinh). 2023 Jun 29;10(26):2302688. doi: 10.1002/advs.202302688 (PMC10502665; doi:10.1002/advs.202302688)
Supplement: Supplementary file 1 — Supporting Information [file ADVS-10-2302688-s001.pdf]

ADVANCED SCIENCE
Open Access

Supporting Information

for *Adv. Sci.*, DOI 10.1002/advs.202302688

Gibbs Adsorption and Zener Pinning Enable Mechanically Robust High-Performance Bi_2Te_3-Based Thermoelectric Devices

Chaohua Zhang, Qiangwen Lai, Wu Wang, Xuyang Zhou, Kailiang Lan, Lipeng Hu, Bowen Cai, Matthias Wuttig, Jiaqing He, Fusheng Liu* and Yuan Yu**

Supporting Information

Gibbs Adsorption and Zener Pinning Enable Mechanically Robust High-Performance Bi_2Te_3-based Thermoelectric Devices

Chaohua Zhang, Qiangwen Lai, Wu Wang, Xuyang Zhou, Kailiang Lan, Lipeng Hu, Bowen Cai, Matthias Wuttig, Jiaqing He, Fusheng Liu*, and Yuan Yu**

Prof. C.H. Zhang, Q.W. Lai, K.L. Lan, Prof. L.P. Hu, Prof. F.S. Liu
College of Materials Science and Engineering, Shenzhen Key Laboratory of Special Functional Materials, Shenzhen Engineering Laboratory for Advanced Technology of Ceramics, Guangdong Research Center for Interfacial Engineering of Functional Materials, Institute of Deep Underground Sciences and Green Energy, Shenzhen University, Shenzhen 518060, P. R. China
E-mail: fsliu@szu.edu.cn (F.S. Liu)

Dr. X. Zhou
Department of Microstructure Physics & Alloy Design, Max-Planck-Institut für Eisenforschung GmbH, 40237 Düsseldorf, Germany

Dr. W. Wang, Prof. J.Q. He,
Department of Physics, Southern University of Science and Technology, Shenzhen 518055, P. R. China
E-mail: hejq@sustech.edu.cn (J.Q. He)

Dr. B.W. Cai
Shenzhen Jianju Technology Co., LTD., Shenzhen 518000, P.R. China

Prof. M. Wuttig, Dr. Y. Yu
Institute of Physics (IA), RWTH Aachen University, 52056 Aachen, Germany
E-mail: yu@physik.rwth-aachen.de (Y. Yu)

Prof. M. Wuttig
PGI 10 (Green IT), Forschungszentrum Jülich GmbH, 52428 Jülich, Germany

Theoretical modeling of device performance

The output power (P) of the thermoelectric (TE) module is calculated as:

$$P = (V_{oc} - R_{in}I)\, I$$

where the V_{oc} is open-circuit voltage, R_{in} is the internal resistance of the TE module, I is the current.

The open-circuit heat flow (Q_{oc}) of the TE module is calculated as:

$$Q_{OC} = N(\frac{A_p}{H}\overline{\kappa_p} + \frac{A_n}{H}\overline{\kappa_n})$$

Where $\overline{\kappa_p}$ is the average thermal conductivity of p-type TE material, and $\overline{\kappa_n}$ is the average thermal conductivity of n-type TE material.

The input heat at the hot side (Q_{input}) of the module is then calculated as:

$$Q_{input} = Q_{oc} - \frac{R_{in}I^2 + N(\beta_p + \beta_n)T_h I}{2} + N[S_p T_h - S_n T_h]T_h I$$

$$\beta_n = \frac{T_c}{T_h}[S_n T_c - \overline{S_n}] + [\overline{S_n} - S_n T_h]$$

$$\beta_p = \frac{T_c}{T_h}[\overline{S_p} - S_p T_c] + [S_p T_h - \overline{S_p}]$$

where β_p and β_n are combined coefficients of p-type and n-type materials, respectively.

Finally, the conversion efficiency (η) of the module can be estimated by:

$$\eta = \frac{P}{Q_{input}} = \frac{P}{Q + P}$$

where Q is the heat flow from the cold side of the TE module.

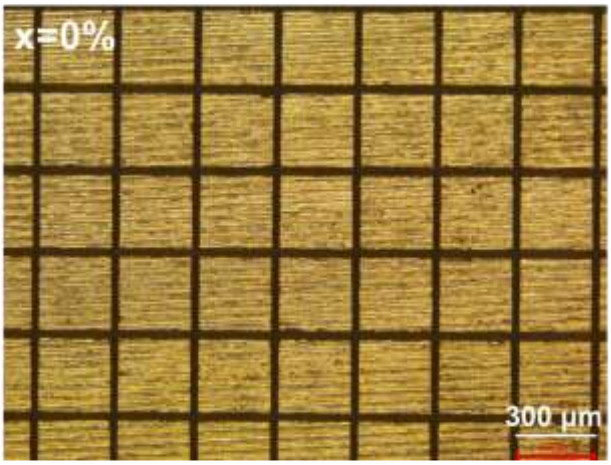

Figure S1. Optical images of the sample $(Bi_{0.5}Sb_{1.5}Te_3)_{1-x}(MgB_2)_x$ with x=0% after cutting into small bars with the size of ~300×300×200 μm. The yield of complete bars is around 100%.

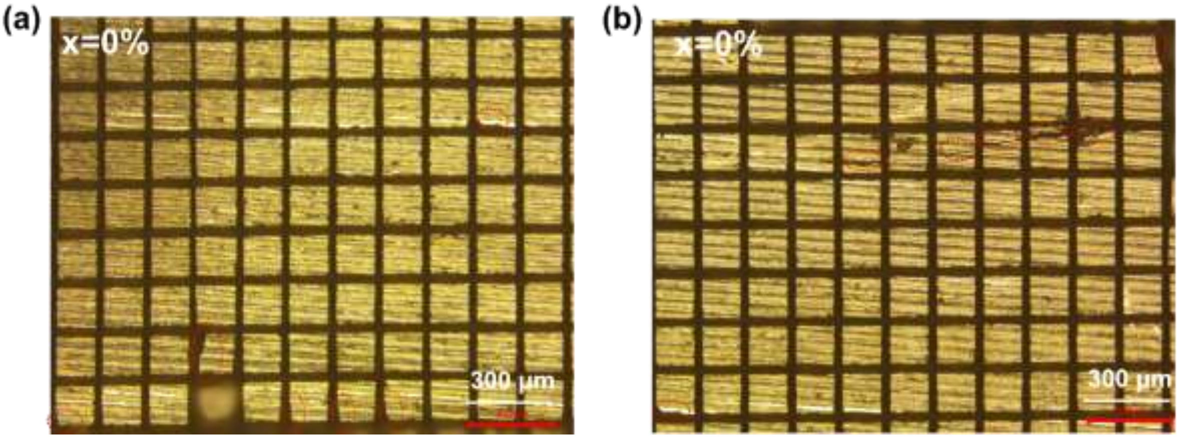

Figure S2. (a)-(b) Optical images of the sample $(Bi_{0.5}Sb_{1.5}Te_3)_{1-x}(MgB_2)_x$ with x=0% after cutting into smaller bars with the size of ~150×150×200 μm. As indicated by those red-dot circles, many obvious missing corners and cracks can be observed. The yield of complete bars is around 92%.

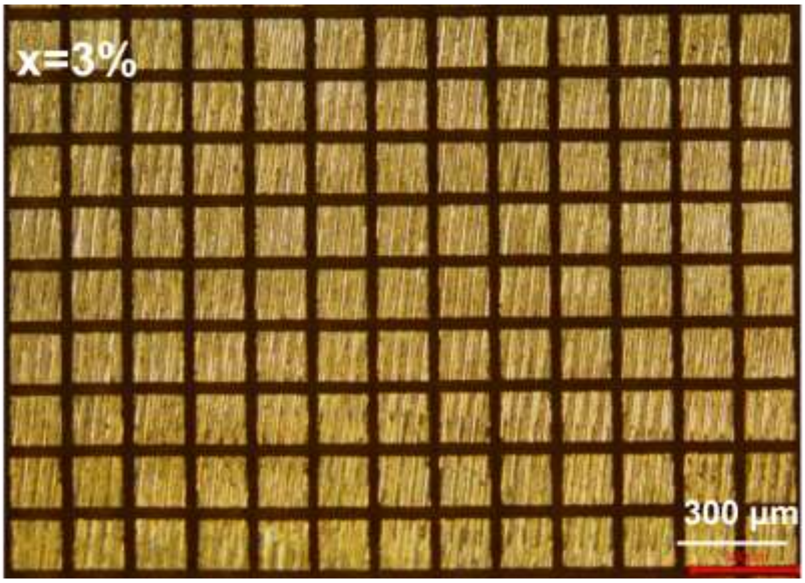

Figure S3. Optical images of the sample $(Bi_{0.5}Sb_{1.5}Te_3)_{1-x}(MgB_2)_x$ with x=3% after cutting into smaller bars with the size of ~150×150×200 μm. No missing corners nor cracks can be observed. The yield of complete bars is around 100%.

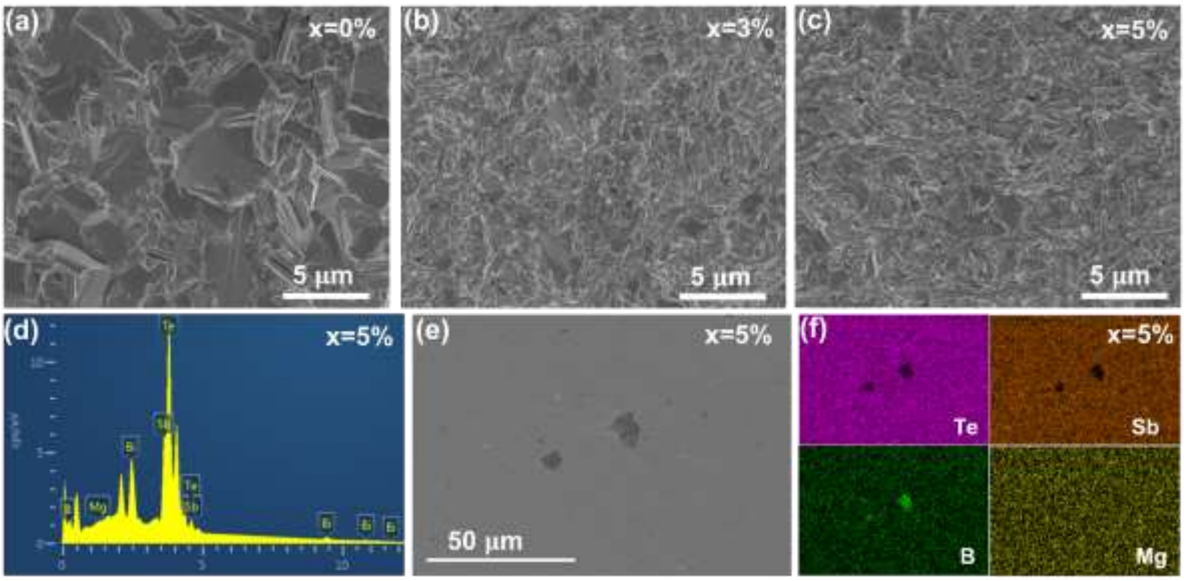

Figure S4. SEM images of the fracture morphology of the $(Bi_{0.5}Sb_{1.5}Te_3)_{1-x}(MgB_2)_x$ alloys with different composition x: **(a)** x=0%, **(b)** x=3%, **(c)** x=5%. **(d)** EDS characterizations of the x-5% sample. **(e)** SEM image of the polished surface for the x=5% sample and **(f)** the corresponding EDS mapping images of Te, Sb, B and Mg.

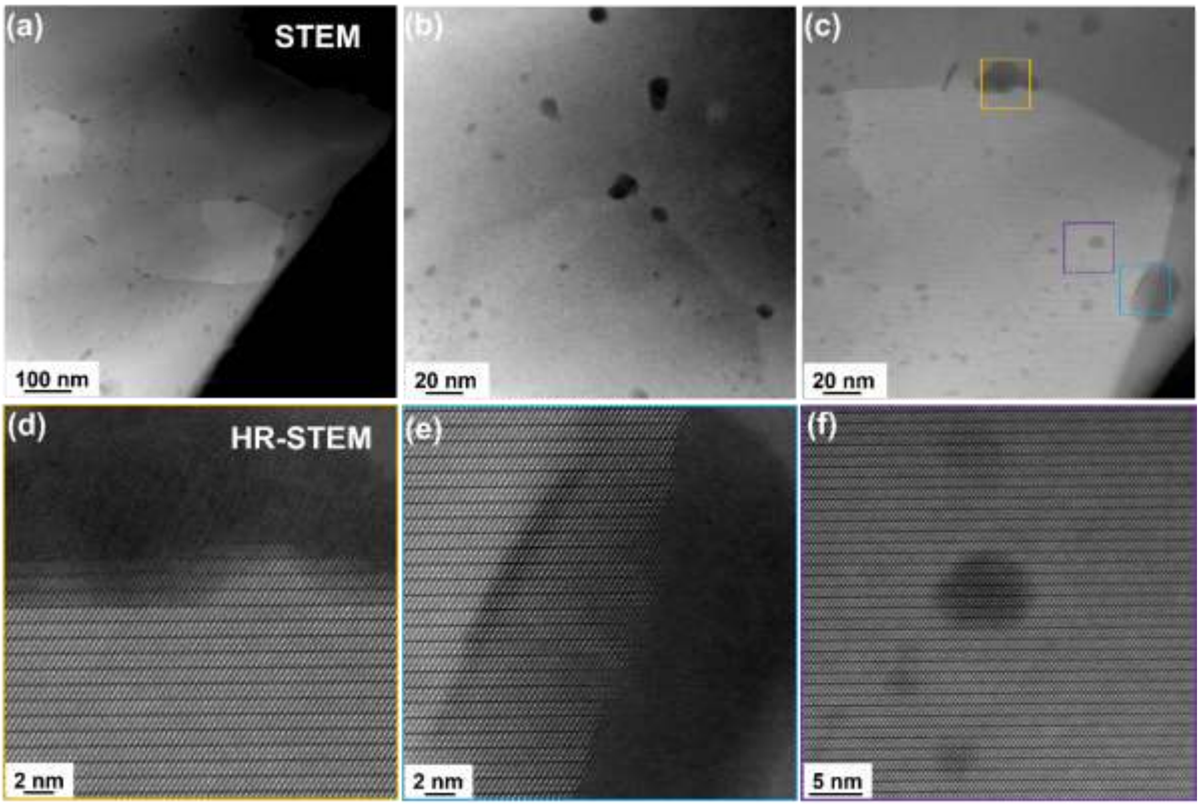

Figure S5. Microstructure characterizations of sample $(Bi_{0.5}Sb_{1.5}Te_3)_{1-x}(MgB_2)_x$ with x=5% by TEM. (a)-(c) Low-magnification TEM image, showing abundant nanoclusters both within grains and along grain boundaries. **(d)-(f)** High-resolution TEM image of different nanoclusters as marked in (c).

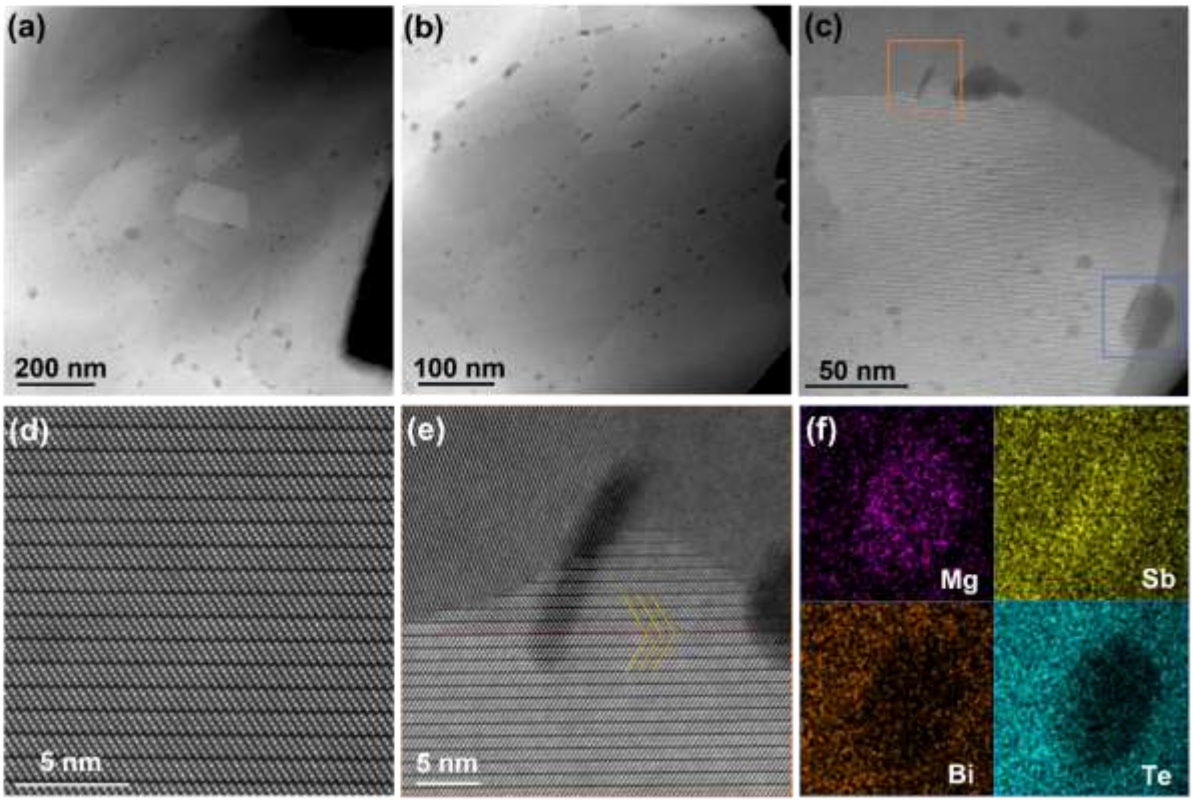

Figure S6. Microstructure characterizations of sample $(Bi_{0.5}Sb_{1.5}Te_3)_{1-x}(MgB_2)_x$ with x=5% by TEM. (a)-(c) Typical low-resolution TEM images in different locations and magnifications. **(d)** Typical high-resolution TEM image within a grain, demonstrating the typical layered structures. **(e)** High-resolution TEM image near a grain boundary marked in (c), showing a twin boundary. **(f)** Elemental mapping images of Mg, Sb, Bi, and Te for a marked region in (c) that include a nanoprecipitate near grain boundary.

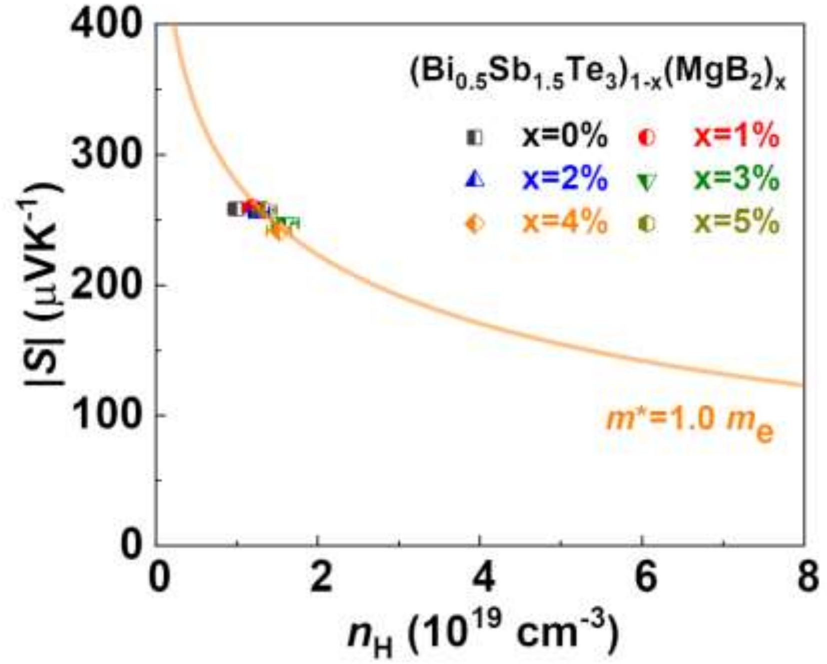

Figure S7. Carrier-density-dependent Seebeck coefficient at 300 K for the samples of $(Bi_{0.5}Sb_{1.5}Te_3)_{1-x}(MgB_2)_x$ with different x. The solid line is calculated based on the single parabolic band model with m^* set as 1.0 m_e.

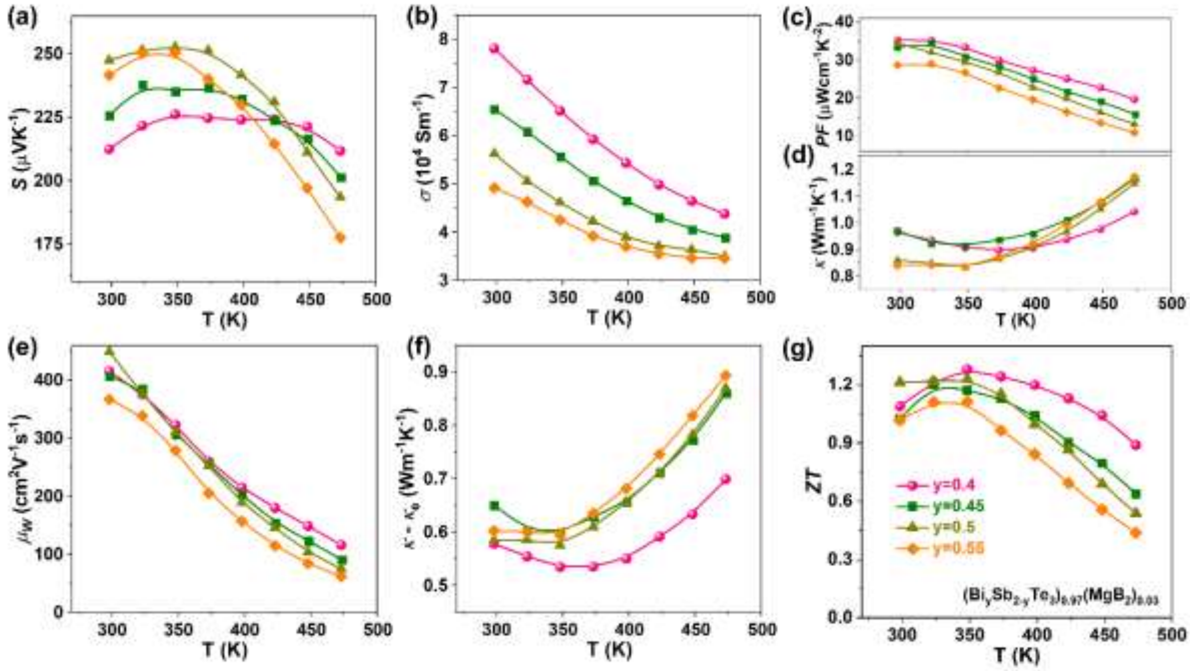

Figure S8. Temperature-dependent **(a)** Seebeck coefficient *S*, **(b)** electric conductivity σ, **(c)** power factor *PF*, **(d)** total thermal conductivity κ, **(e)** weighted mobility μ_w, **(f)** lattice thermal conductivity κ-κ_e, and **(g)** *ZT* of $(Bi_ySb_{2-y}Te_3)_{0.97}(MgB_2)_{0.03}$ with different Bi/Sb ratio.

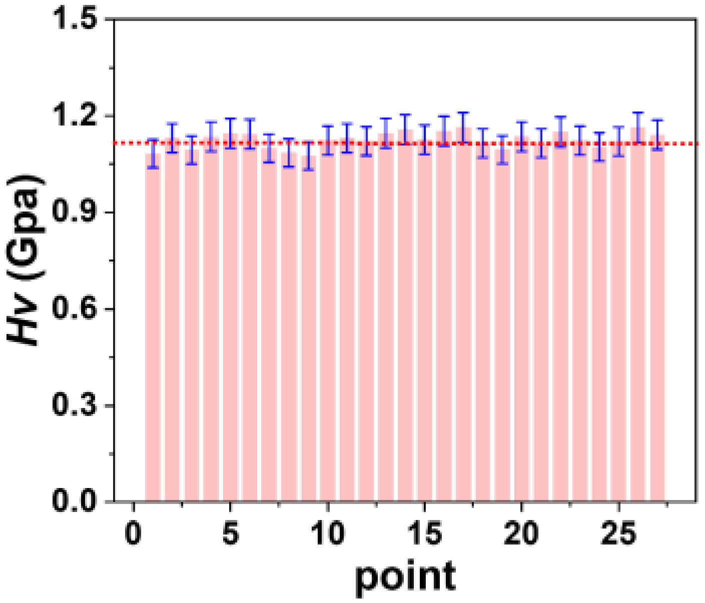

Figure S9. Vickers hardness of a sintered $(Bi_{0.4}Sb_{1.6}Te_3)_{0.97}(MgB_2)_{0.03}$ pellet taken at 27 different locations. Considering the measurement errors of ~4%, the hardness at different locations is nearly at the same level, which demonstrates the uniformity and repeatability of our samples.

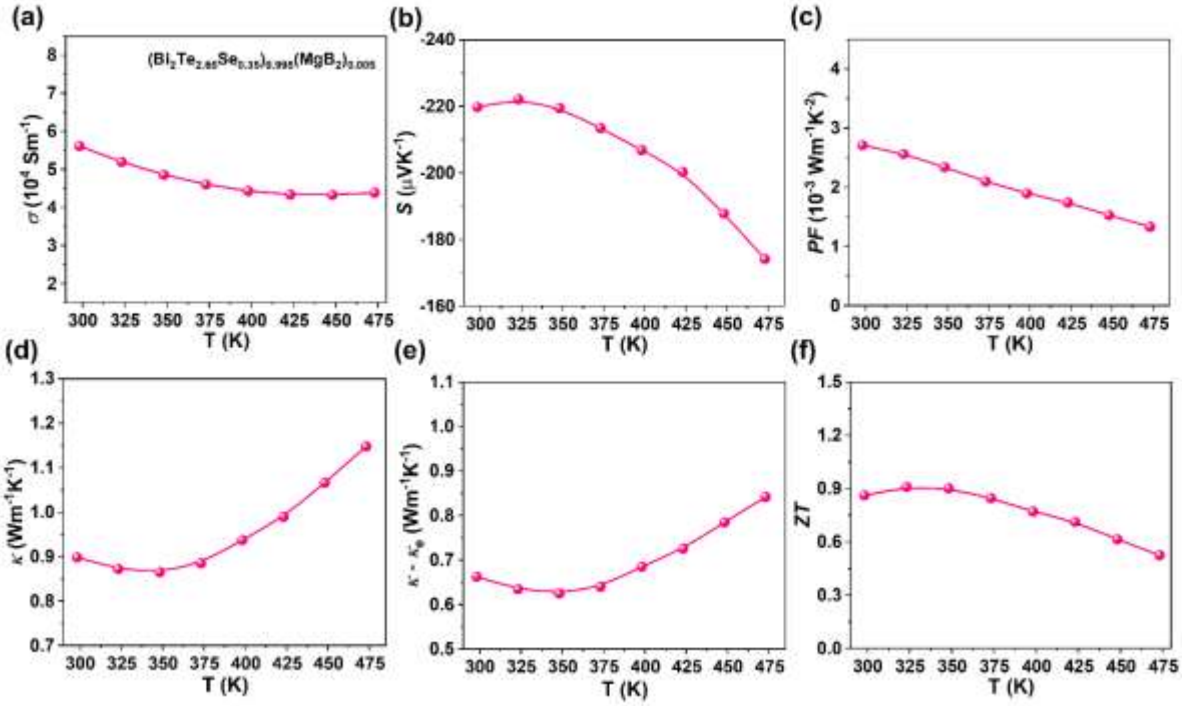

Figure S10. Temperature-dependent **(a)** electric conductivity σ, **(b)** Seebeck coefficient S, **(c)** power factor PF, **(d)** total thermal conductivity κ, **(e)** lattice thermal conductivity κ-κ_e, and **(f)** ZT of the n-type $(Bi_2Te_{2.65}Se_{0.35})_{0.995}(MgB_2)_{0.005}$.

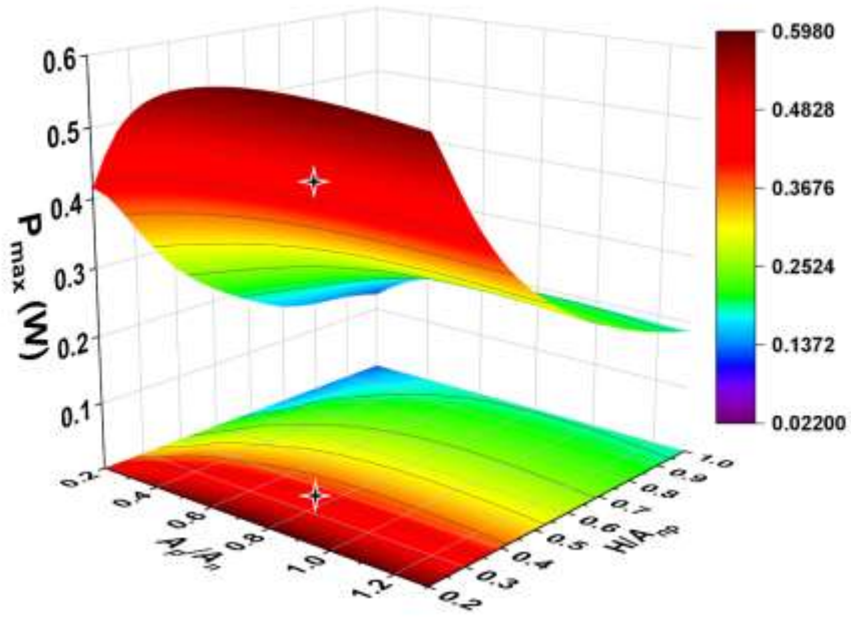

Figure S11. Theoretical modeling of the maximum output power (P) at temperature differences (ΔT) of 215 K (cold-site temperature fixed at 283 K), where the section area ratio of p-type versus n-type leg (A_p/A_n) and the ratio of leg height versus total section area (H/A_{np}) are varied.

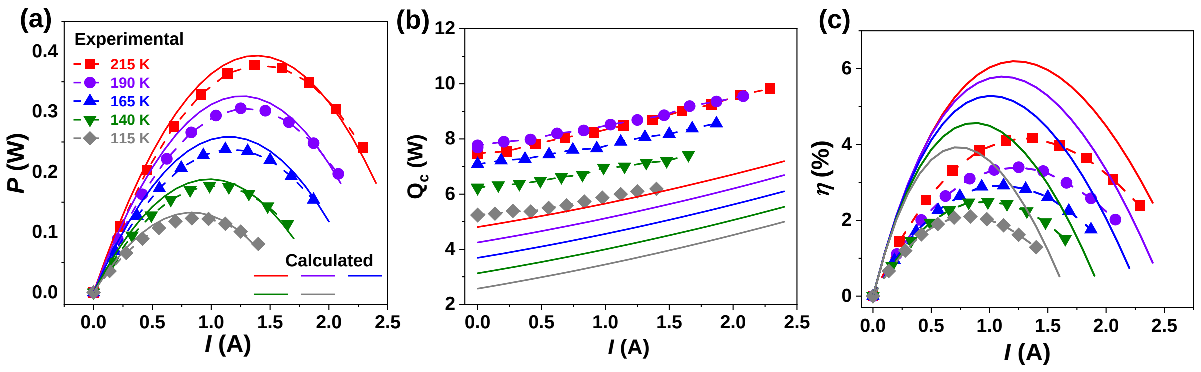

Figure S12. Current-dependent **(a)** output power P, **(b)** heat flow at cold site Q_c, and **(c)** η of the TE device under different ΔT. The solid lines in these figures are corresponding theoretical values, which are calculated based on the experimental parameters of the thermoelectric materials as well as the measured contacting resistance.

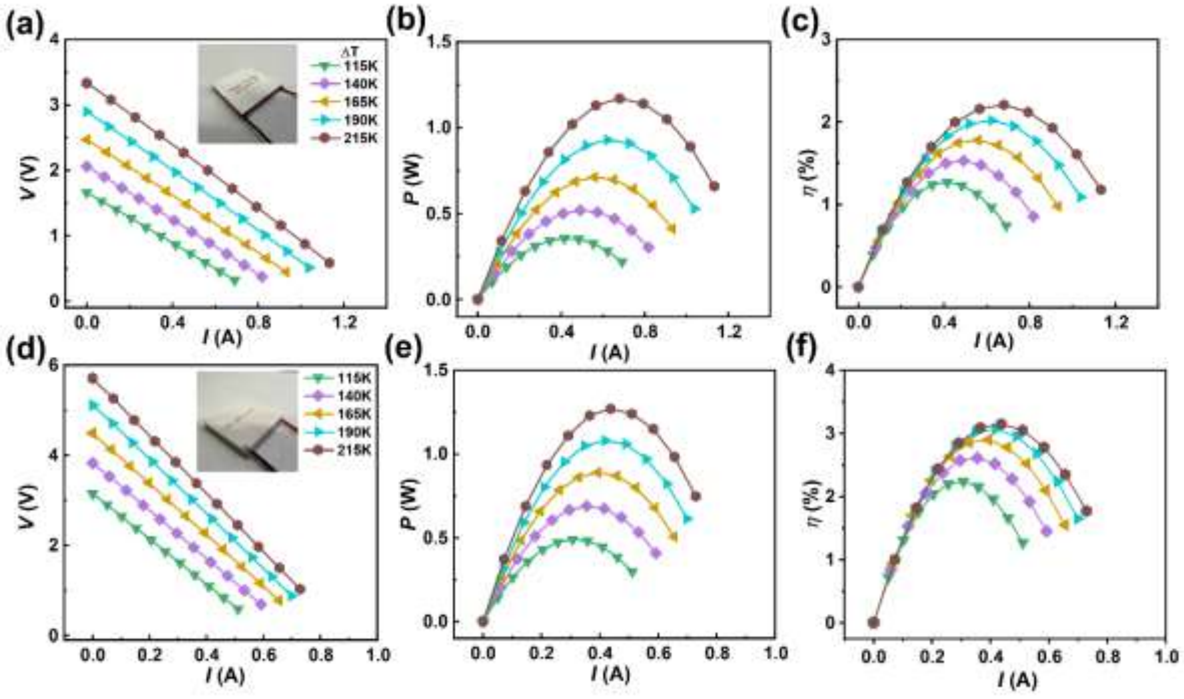

Figure S13. Thermoelectric module characterizations of three purchased commercial TE devices from different companies using our PEM-2 instrument. Current-dependent **(a)** output voltage (*V*), **(b)** output power (*P*) and **(e)** energy conversion efficiency (η) of the CTE-1 device under different temperature differences (ΔT). Corresponding device characterizations for **(d)-(f)** CET-2. The optical images of the three commercial TE devices CTE-1 and CTE-2 are displayed in the inset of (a) and (d), respectively.
